# Following prevalence of myopia in a large Swiss military cohort over the last decade: where is the European “myopia boom”?

**DOI:** 10.1007/s00417-024-06467-0

**Published:** 2024-04-05

**Authors:** Leila Sara Eppenberger, Gregor P. Jaggi, Margarita G. Todorova, Jürg Messerli, Veit Sturm

**Affiliations:** 1 Health Sciences and Technology, ETH Zurich, Raemistrasse 101, 8092 Zurich, ZH Switzerland; 2 Eye Clinic, Cantonal Hospital of Lucerne, Spitalstrasse, 6000 Lucerne, LU Switzerland; 3 Singapore Eye Research Institute, Singapore National Eye Center, 20 College Rd, Singapore, 169856 Singapore; 4 Faculty of Medicine, University of Zurich, Pestalozzistrasse 3, 8032 Zurich, ZH Switzerland; 5 Eye Clinic, Cantonal Hospital of St. Gallen, Rorschacher Str. 95, 9007 St. Gallen, SG Switzerland; 6 Eye Clinic, University Hospital of Basel, Mittlere Strasse 91, 4031 Basel, BS Switzerland; 7 Health Department, Swiss Armed Forces, Ittigen, Switzerland

**Keywords:** Europe, Myopia prevalence, Military conscripts, Myopia boom, Swiss military cohort

## Abstract

**Purpose:**

Myopia prevalence is increasing globally, with the highest rates found in Asia. Data from European countries is scarce. We aimed to investigate whether the prevalence of myopia is rising in our meridians.

**Methods:**

Data from male military conscripts for the recruitment period of 2008–2017 were retrospectively analyzed. Year of recruitment, conscripts’ birth year, visual acuity, refractive status (spherical equivalent), and spectacle wear (yes/no) were available.

**Results:**

The dataset contained data of a total of 355,657 male conscripts, who had been recruited in the years 2008 to 2017. The mean number of conscripts per year was 35,566 (MD = 35,440, SD = 1249), reaching a minimum number of 33,998 conscripts in 2017 and a maximum of 37,594 in 2011. Mean age at recruitment was 19.7 years (MD = 19.0 years, SD = 1.1 years). Overall, the number of conscripts wearing spectacles remained stable over the observation time; on average 29.6% (*n* = 10,540; MD = 10,472; SD = 492) of conscripts wore glasses at recruitment. Of 21.8% (*n* = 77,698) of conscripts, data on the refractive status was available: The mean spherical equivalent for both right and left eyes was -2.3D (MD = -2 D, SD = 2.4 D). No decrease in mean spherical equivalent per recruitment year was noted over the observation period. Estimated myopia prevalence reached an average of 27.5% (SD = 0.8%) and did not increase during the observation period.

**Conclusion:**

In summary, no change in spherical equivalent refractive errors of male Swiss army conscripts was found for the years 2008–2017. Equally, the percentage of spectacle wearers (MN = 29.6%) and estimated myopia prevalence (MN = 27.5%) did not significantly increase during the observation time.

**Trial registration**: BASEC 2019-00060 (18/01/2019)

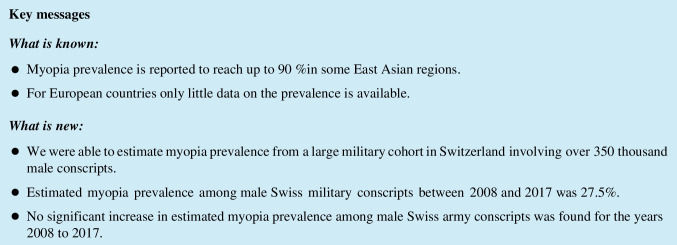

## Introduction

With almost an exponential increase in prevalence in certain areas of the world over the last few decades, myopia and its complications have become a significant global public health and socioeconomic challenge with an epidemic character. Current estimations indicate that approximately a third of the world population is myopic. Noteworthy, there are significant geographical differences in the prevalence of myopia [1]. Myopia, defined as an objective refractive error with a spherical equivalent (SE) of ≤ –0.50 diopters (D), reaches prevalence rates of up to 80–90% in some East Asian regions. In the same regions, high myopia (HM), comprising conditions with a SE of ≤ –5.00 D in either eye, accounts for up to 20% [2–5]. Currently, about 30% of the global population are affected by myopia; the global prevalence is expected to rise to a considerable 50% of the global population by 2050 [1]. Often myopia is considered to be benign and easily correctable, but especially when it increases in severity, its complication can lead to severe vision impairment and reduced quality of life. Especially, HM is known to be associated with an increased risk of irreversible vision impairment or even blindness due to pathological changes in the eye which are still challenging and costly to manage [6].

As mentioned above, the prevalence of myopia and HM in urbanized East Asian regions are extremely high. From population studies using cycloplegic refraction, it is known that the prevalence of myopia among 12-year-old children in different countries ranges from 6.0% in Cambodia, to 7.4% in New Delhi, to 11.9% in Australia, to 17.7% in Northern Ireland, and 20.0% in the United States to 49.7% and 53.1% in China and Hong Kong, respectively [7–13]. Noticeably, myopia prevalence in school children reaches an even higher percentage of 62.0% in Singapore [14]. Although myopia prevalence is highest in some urbanized areas of aforementioned east Asian countries, there is still a large variance. This is most likely due to ethnic and above all, behavioral and educational differences, which lead to an acceleration of myopia development, especially if these are present at vulnerable developmental age [15–21].

Also in young adults, the prevalence of myopia and HM varies between different ethnicity and geography. A population-based prevalence survey in Israel conducted on 16–22-year-olds during the years 1990 to 2002 revealed an overall increase in myopia prevalence using non-cycloplegic autorefraction from 20.3% to 28.3% [22]. In Australia, the prevalence in subjects aged 19–22 years was 20.4% [23]. Among Danish conscripts, the prevalence of myopia and HM was lower, with 12.8% and 0.3%, respectively [24]. In Norway, 35% of 20–25-year-olds were reported to be myopic [25]. The pooled estimated prevalence in Europe was 27% (95% CI = 22.4–31.6) [3]. In the United States, the average myopic prevalence rate was 27.7% in young adults aged 18–24 years [26], but other studies reported a range from 16 to 34%, depending on the region [3]. On the other hand, the myopia prevalence in urbanized East Asian countries was reported to reach up to 90% for myopia and up to 20% for HM [4, 5].

Since data on myopia prevalence in European countries is scarce, our goal was to provide information about the situation in our latitudes over the last decade. Our aim was to investigate and estimate the prevalence of myopia in a large Swiss military cohort for the years 2008–2017, as well as to evaluate whether an increase could be detected over this time.

## Methods

After reaching out to the Swiss Armed Forces, an opportunity to analyze data on vision and refractive data from a large cohort of young Swiss male conscripts arose.

In Switzerland, every Swiss male, aged 18–30 years, is required to serve in the military service or civilian protection. A summons is sent to every single Swiss male in the year he turns 18 and he must attend a recruitment event at the latest the year he turns 24 years. For Swiss women, the military service is voluntary. The recruitment process takes two to three days and involves among others a detailed medical examination, including a comprehensive eye examination [27, 28].

Here, we investigated a large dataset provided by the Swiss Armed Forces, which included data collected during the recruitment of military conscripts who had undergone their recruitment process in the years between 2008 and 2017. This cross-sectional retrospective cohort study was conducted in accordance with the tenets of the Declaration of Helsinki. The study protocol was approved by the regional ethics committee (BASEC 2019–00060).

In Switzerland, there are six recruitment centers, where according to a structured protocol young men are tested, regarding their eligibility to serve in the military service. The test battery for vision and eye examination during recruitment involves visual acuity (VA) tested with the Pflüger optotypes (analogous to the Snellen-E-Chart); a VA maximum of 2.0 can be reached. VA without correction and additionally, if available, with spectacles is tested by a trained staff. The VA values, as well as those of the spectacles’ prescription, are transferred to the Swiss Armed Forces’ electronic medical chart, provided that the prescription is less than 1 year old. If the prescription is not up to date (> 1 year) and/or if the VA requirements are not met, a new refraction is taken by the in-house optician.

From the electronic medical chart, an anonymized dataset containing the following variables was extracted and transferred via a protected channel for further analysis: year of recruitment, the conscripts’ birth year, spectacles wearing (yes/no), VA with or without correction, and refraction per eye, as well as information on color vision and stereopsis (Lang Stereotest).

## Statistical analysis

Statistical analysis was primarily descriptive. Based on the available refractive values and spectacles-wearing (yes/no) myopia, prevalence per recruitment year was estimated. Wilcoxon Signed Rank Sum test, Student’s t-test, ANOVA (one-way/two-way), and chi-squared test were used for comparisons of the number of spectacles-wearing and myopic conscripts of every recruitment year. The Mann–Kendall test was applied to evaluate trends in rates from 2008 to 2017. *P* values < 0.05 were considered significant. Statistical analysis was performed in R version 4.3.3 (2024–02-29).

## Results

After data cleaning, which involved excluding a few cases (*n* = 773, 0.2%) with age outside of the range of 18 to 25 years at recruitment (*n* = 444) or extreme and unplausible refractive or VA values (*n* = 329), as well as the data of all female conscripts (*n* = 2296); the dataset contained data of a total of 355,657 male conscripts, who had undergone recruitment process in the years 2008–2017.

**Fig. 1:**
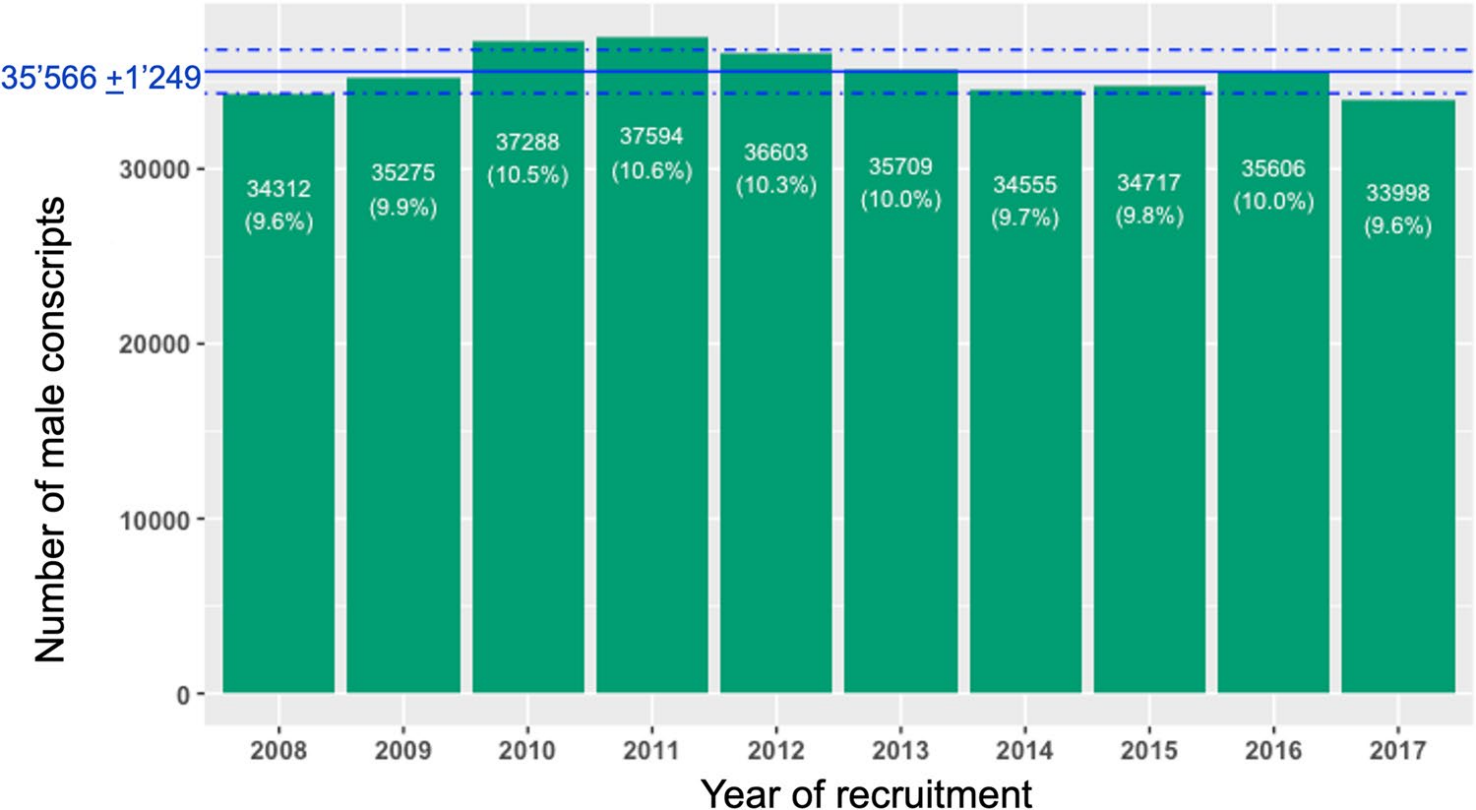
This histogram shows the absolute number of male conscripts included in this study per recruitment year. The median number of conscripts was 35,566 per year. Comparable cases were analyzed per year

Figure 1 shows the distribution of the total number of conscripts per year. The mean number of conscripts per year was 35,566 (MD = 35,440, SD = 1249), reaching a minimum of 33,998 conscripts in 2017 and a maximum of 37,594 in 2011. There was no statistical change in number of recruits per year from 2008 to 2017 (tau = -0.24, *p* = 0.4). The mean age at recruitment was 19.7 years (MD = 19.0y, SD = 1.1y), and it was statistically significantly lower (*p* < 0.001), but only minimally in absolute values, for the recruitment years of 2014–2017 (MN = 19.6y) compared with the recruitment years 2008–2013 (MN = 19.8y).

**Fig. 2:**
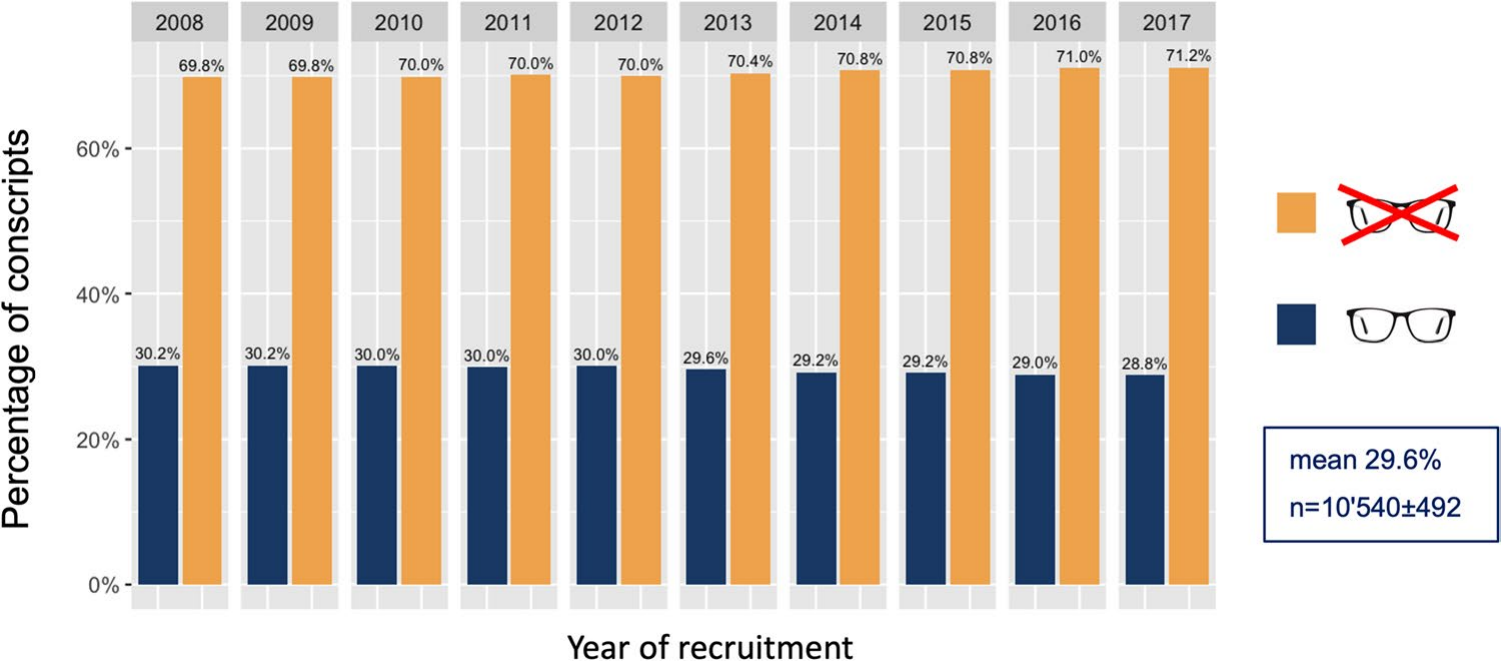
The mean percentage of conscripts wearing spectacles in the time 2008–2017 was 29.6%; there were slightly more conscripts wearing spectacles in the year 2008, compared to 2017

Overall, on average 29.6% (*n* = 10,540; MD = 10,472; SD = 492) of conscripts wore glasses (or contact lenses) at recruitment (see Fig. 2) ranging from 30.2% in 2008 to 28.8% in 2017. This downward trend in the annual number of conscripts wearing glasses did not reach statistical significance according to Mann–Kendall test (tau = -0.47, *p* value = 0.07).

**Fig. 3:**
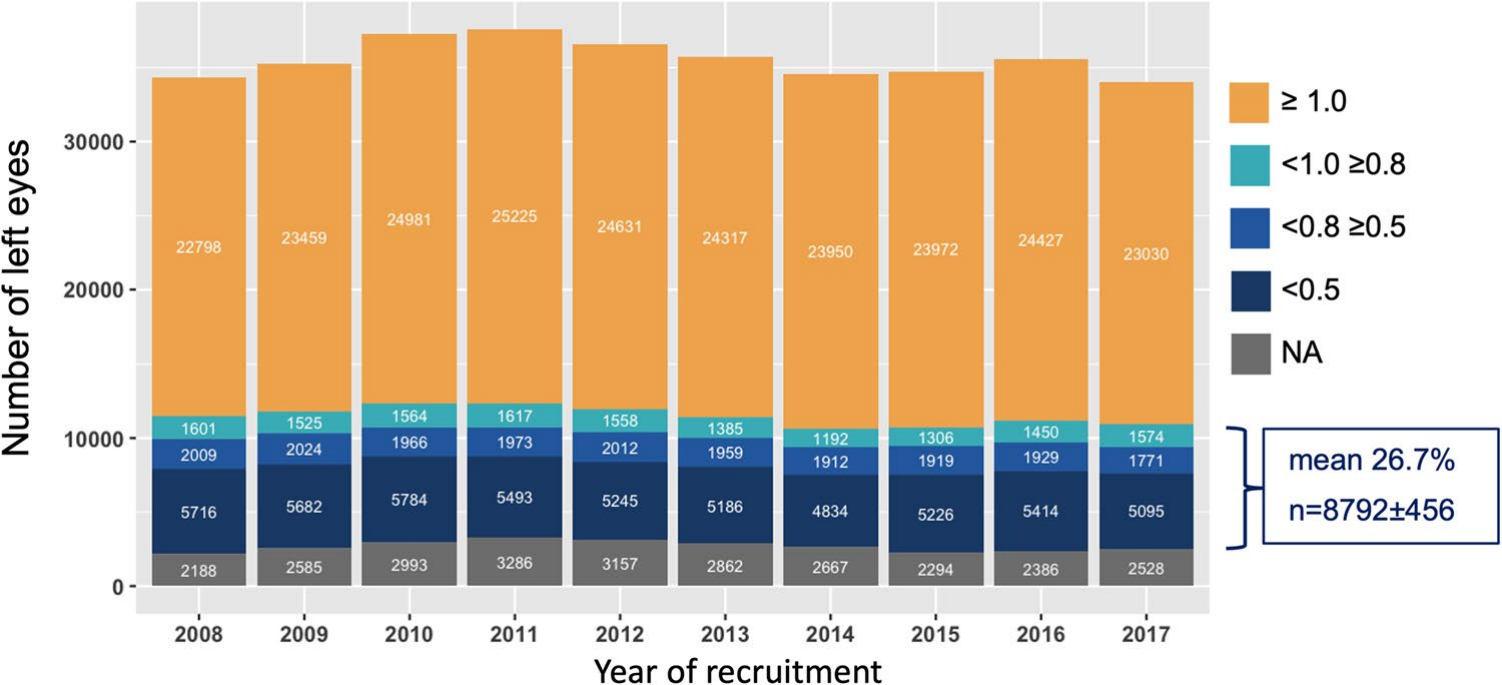
Similar numbers of conscripts with left eyes with full corrected or uncorrected visual acuity (VA) (yellow), i.e., ≥ 1.0 decimal were observable in the 10 years investigated in this study. Conscripts achieving a VA of < 1.0– ≥ 0.8 in their left eyes are colored in turquoise, conscripts with a VA in their left eyes of < 0.8– ≥ 0.5 are marked in blue, and those having very low VA are marked in dark blue

The overall uncorrected VA (see Fig. 3; here only data from left eyes is shown, but very similar distributions are found in right eyes) were stable over the 10 years between 2008 and 2017, with comparable proportions of conscripts with a VA equal or above 1.0 per year. Statistically, there were some small but significant differences between the percentages of conscripts with full VA (X-squared = 204.68, df = 9, *p* value < 0.001): In 2008, the lowest percentage of 71.0% of full VA was recorded, the highest proportion with 75.1% in 2014. When looking at the time-dependent variation of the number of cases with full VA, however, there was no statistically significant up- or downward trend (tau = 0.467, *p* = 0.07). Data on VA was missing for an average of 7.6% of conscripts; this rate was similarly distributed over the observation period (tau = -0.11, *p* = 0.72). The lowest missing data rate was 6.6% in 2015 and reached 8.7% in 2011.

**Fig. 4:**
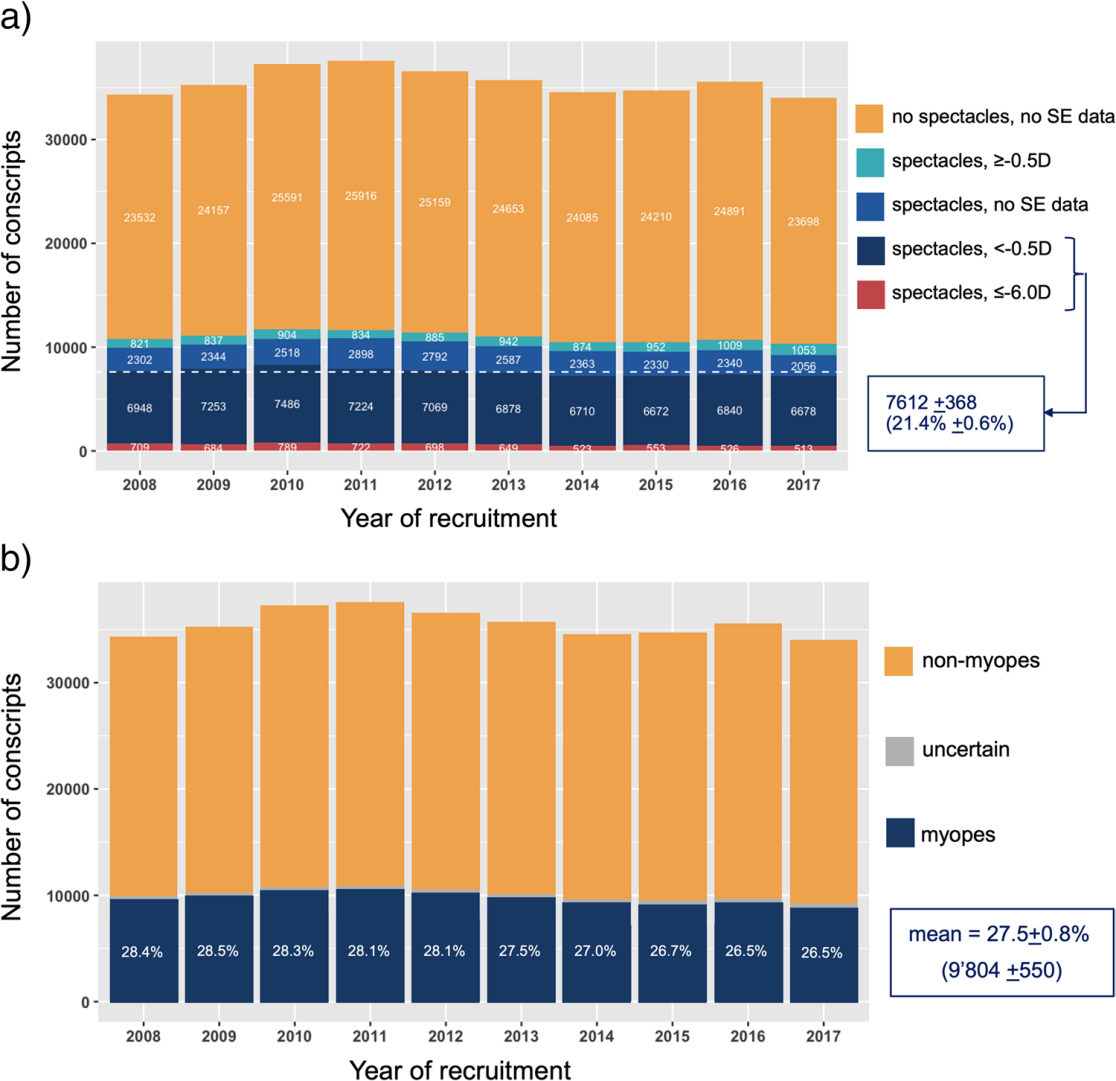
Estimating myopia prevalence from spectacle-wearing status and available data on spherical equivalent (SE). **a** Absolute numbers of conscripts per year with information on spectacle wearing and if available SE: in yellow conscripts without spectacles; emmetropic and hyperopic conscripts in turquoise; conscripts wearing spectacles but with missing data on SE in blue; myopic conscripts with SE of < -0.5D to > -6.0D in dark blue; and highly myopic conscripts with SE =  < -6.0D. Median total number and percentage of myopic conscripts are displayed in the box. **b** Assuming a ratio of 9:1 myopes (blue and red) to emmetropes and hyperopes (turquoise) for the subgroup of spectacle wearers with unknown SE, yearly myopia prevalence is estimated. In yellow, the non-myopes (sum of number of conscripts without spectacles, emmetropes and hyperopes), in blue estimated myopes (sum of myopes, high myopes, and 90% of conscripts with spectacles but unknown SE), and grey represents the estimated “uncertain” cases (equal to 10% of conscripts with spectacles but unknown SE, see blue category in Fig. 4a)

For the estimation of myopia prevalence, we defined the following groups of conscripts (see Fig. 4): all non-spectacle wearers, without data on SE; spectacle wearers with SE ≥ -0.5D in both eyes, so emmetropes and hyperopes. Myopes were defined as spectacle wearers with < -0.5D SE in at least one eye and high myopes with SE of ≤ -6.0D in at least one eye. The ratio of myopes to emmetropes/hyperopes in the group of spectacles-wearing conscripts with known SE was approximately 9:1 for all ten recruitment years. We assumed the same 9:1 proportion of myopes to emmetropes/hyperopes for the group of spectacles-wearing conscripts without available SE data and then added these 90% of the “uncertain” spectacle wearers to the myopes with known SE. This resulted in an estimated yearly myopia prevalence of 27.5%. The range of estimated myopia prevalence varied from 26.5% in 2016 and 2017 to 28.5% in 2009 (X-squared = 50.89, df = 9, *p* value < 0.001). There was no increase in estimated myopia year-wise prevalence. Mann–Kendall test detected a downward trend of estimated myopia cases (tau = -0.51, *p* = 0.05).

From the 21.8% (*n* = 77,365) of conscripts, with available refractive status of their left eyes, the overall mean SE was -2.3D (MD = -2D, SD = 2.4D). Overall, there was no decrease in mean SE for the recruitment years 2008–2017. Statistically, there was a small, but significant increase in mean SE, with a visible change from -2.4D in 2008 compared with -2.0D in 2017 (tau = 0.733, *p* = 0.004). When analyzing the in-between years differences, no statistically significant differences were found (with only one exception for the comparison of mean SE of 2015 and 2016, with mean SE from left eyes of -2.15D and -2.06D, respectively (*p* = 0.03)).

Similarly, we found the mean SE for right eyes to be -2.3D (MD = -2D, SD = 2.4D; *n* = 77,698), with no decrease between 2008 to 2017. Comparable to left eyes, there was an upward trend in the mean SE over the investigated time (tau = 0.733, *p* < 0.004).

We further explored whether the observed significantly lower mean age at recruitment for the years 2014 to 2017 compared with the years 2008–2013 could explain the slight but statistically significant increase in mean SE. We found that indeed older conscripts (22 years) had statistically lower mean SE compared to younger conscripts (19 years) (*p* = 0.02) of whom more were present in the more recent recruitment years 2014–2017.

## Discussion

The main strength of this analysis is the enormous and representative number of conscripts included in the cohort. To the best of our knowledge, there is only one comparable study regarding statistical power on myopia prevalence in Europeans [29]. Another strength is the fact that the recruitment process is standardized, and hence the data on VA and refraction is similarly acquired for every military conscript included. Furthermore, the data is electronically registered in a central database, easily extractable by the Swiss Armed Forces data management. The data cleaning process demonstrated the high data quality, as only a neglectable percentage (0.2%, *n* = 773) of cases had to be excluded, because the conscripts’ age did not fall into the predefined range of 18–25 years, or due to unrealistically high values in their refraction or VA, which we assumed to be most likely documentation errors. In Switzerland, military service is compulsory, and almost every man of Swiss nationality receives an invitation to an obligatory recruitment; hence, the analyzed cohort seems to represent the young male Swiss population well. Compared with the data openly accessible by the Swiss Armed Forces on the yearly examined conscripts numbers, we realized that in the analyzed dataset for the observation time of 2008–2017, the absolute numbers were only slightly lower and hence representative [30–39].

As to the limitations of this military conscripts’ cohort study: First, the analyzed data represents a *male* population. According to the literature, myopia prevalence in females is usually slightly higher compared with the one in males [3]. In the original dataset provided by the Swiss Armed Forces, the number of female conscripts was very small and variable each year, necessitating the analysis to be restricted to males. This because for Swiss women military service is voluntary. Secondly, no information on the progression of refractive error on an individual level was available, due to the cross-sectional nature of the recruitment process. Another noteworthy limitation is that information on refraction was only present for spectacles-wearing conscripts and based on non-cycloplegic measurements. Although there was no standardized procedure for the refractive measurements performed by the opticians, we assume the values to be adequate as they are used to fabricate personalized, costly military spectacles, and lenses for the protection masks.

Overall, the percentage of spectacle wearers among male Swiss army conscripts remained stable during the last decade. We found an estimated average myopia prevalence from 2008 to 2017 of 27.5%. There was a small, but statistically significant decrease in the number of spectacles-wearing conscripts and estimated myopia cases over time. In analogy, the mean SE in the years 2008–2013 was slightly lower compared to 2014–2017. These unexpected findings could be explained by the slightly lower age of conscripts in the more recent years. Nevertheless, in summary, we found no increase in estimated prevalence of myopia among male Swiss military conscripts for the period of 2008–2017.

Our estimated myopia prevalence was a bit higher, but still comparable in percentage with the little previously published data: Yang et al. investigated 18-year-old Austrian conscripts and found an increase of myopia prevalence from 18% to 24.4% over 35 years from 1983 to 2017 [29]. We could not observe an increase in myopia prevalence over time, possibly due to the relatively smaller period of 10 years investigated. However, reports from older studies with similar cohorts displayed a lower myopia prevalence of about 13% in 2004, but also no increase over time when compared with data from 1882 to 1964 [24]. A similar myopia prevalence of 29.3% was found in Iran, among young adults aged 16–25 years [40]. In a much smaller population-based study from Norway, there were 35.0% myopic young adults aged 20–25 years [25]. Alike to our study, a population-based prevalence survey with a 13-year series (1990–2002) was conducted on young Israeli adults aged 16–22 years. The overall prevalence of myopia and HM (SE <  − 6.0 D) using non-cycloplegic autorefraction measurement was found to increase from 20.3% and 1.7% in 1990 to 28.3% and 2.05% in 2002, respectively [41]. Depending on the investigated time and locations, as well as methods to assess myopia prevalence, the few reports from Europe are disparate. In agreement with our observation of a stable estimated myopia prevalence, in the KiGGS study from Germany, Schuster et al. did not find an increase in myopia cases from 2003 to 2006, compared with 2014–2017 [42, 43]. In summary, our findings seem largely in line with the little reported data from Europe and stand quite clearly in contrast to the much higher numbers of myopia cases in some East Asian countries [44].

In the large meta-analysis by Rudnicka et al. investigating time trends of myopia in children and adolescents, ethnic differences in age-specific prevalence of myopia were found, with higher and earlier increase of myopia in East Asians. Especially, children from urban environments had 2.6 times the odds of myopia compared to those from rural environments [45]. In our analysis, no information about the ethnicity or the environments in which the conscripts were raised was available. Nevertheless, it can be assumed that the Swiss military conscripts represent a homogenous ethnic group of predominately European descent [46]. As to further environmental/behavioral factors influencing myopia prevalence, the investigated cohort of conscripts aged18–25 years between 2008 to 2017 belonged right about to the generation, who started being more and more exposed to digitalization but most likely was not yet using electronic devices, such as mobile phones and tablets at an infant age. The younger generation which is subject to new and possibly hazardous environmental and behavioral changes regarding, for example, practices in schools, e.g., increased screen and near-work, changes in lighting, and time spent outdoors, is not included here. Thus, it cannot be excluded that if more recent data from the post-COVID-19 pandemic time were to be analyzed, an increase in myopia in European latitudes could be detected. Future research and further comparison with other large population-based data, ideally from more recent years, is necessary and of great value.

## Conclusion

In summary, no decrease in spherical equivalent in male Swiss military conscripts was found for the years 2008 to 2017. Equally, the percentage of spectacle wearers (mean 29.6%) and estimated myopia prevalence (mean 27.5%) did not significantly increase during the observation time.

## Data Availability

The data will be made available upon request to the corresponding authors and after consent from the Swiss Armed Forces.

## Code availability

The code will be made available upon request to the corresponding authors and after consent from the Swiss Armed Forces.

## References

[1] Holden BA, Fricke TR, Wilson DA et al (2016) Global prevalence of myopia and high myopia and temporal trends from 2000 through 2050. Ophthalmology 123:1036–1042. 10.1016/j.ophtha.2016.01.006 26875007 10.1016/j.ophtha.2016.01.006

[2] Xiong S, Sankaridurg P, Naduvilath T et al (2017) Time spent in outdoor activities in relation to myopia prevention and control: a meta-analysis and systematic review. Acta Ophthalmol 95:551–566. 10.1111/aos.13403 28251836 10.1111/aos.13403 PMC5599950

[3] Hashemi H, Fotouhi A, Yekta A et al (2018) Global and regional estimates of prevalence of refractive errors: systematic review and meta-analysis. J Curr Ophthalmol 30:3–22. 10.1016/j.joco.2017.08.009 29564404 10.1016/j.joco.2017.08.009 PMC5859285

[4] Lin L, Shih Y, Hsiao C, Chen C (2004) Prevalence of myopia in Taiwanese schoolchildren: 1983 to 2000. Ann Acad Med Singap 33:27–33. 10.47102/annals-acadmedsg.V33N1p27 15008558 10.47102/annals-acadmedsg.V33N1p27

[5] Jung S-K, Lee JH, Kakizaki H, Jee D (2012) Prevalence of myopia and its association with body stature and educational level in 19-year-old male conscripts in Seoul, South Korea. Invest Ophthalmol Vis Sci 53:5579–5583. 10.1167/iovs.12-10106 22836765 10.1167/iovs.12-10106

[6] Ang M, Wong TY (2020) Updates on myopia: a clinical perspective. Springer Singapore, Singapore

[7] Ip JM, Huynh SC, Robaei D et al (2008) Ethnic differences in refraction and ocular biometry in a population-based sample of 11–15-year-old Australian children. Eye (Lond) 22:649–656. 10.1038/sj.eye.6702701 17277756 10.1038/sj.eye.6702701

[8] Fan DSP, Lam DSC, Lam RF et al (2004) Prevalence, incidence, and progression of myopia of school children in Hong Kong. Invest Ophthalmol Vis Sci 45:1071–1075. 10.1167/iovs.03-1151 15037570 10.1167/iovs.03-1151

[9] He M, Zeng J, Liu Y et al (2004) Refractive error and visual impairment in urban children in southern china. Invest Ophthalmol Vis Sci 45:793–799. 10.1167/iovs.03-1051 14985292 10.1167/iovs.03-1051

[10] Zadnik K (1997) The Glenn A. Fry Award Lecture (1995). Myopia development in childhood. Optom Vis Sci 74:603–608 9323731 10.1097/00006324-199708000-00021

[11] O’Donoghue L, Kapetanankis VV, McClelland JF et al (2015) Risk factors for childhood myopia: findings from the NICER Study. Invest Ophthalmol Vis Sci 56:1524–1530. 10.1167/iovs.14-15549 25655799 10.1167/iovs.14-15549

[12] Gao Z, Meng N, Muecke J et al (2012) Refractive error in school children in an urban and rural setting in Cambodia. Ophthalmic Epidemiol 19:16–22. 10.3109/09286586.2011.632703 22273355 10.3109/09286586.2011.632703

[13] Murthy GVS, Gupta SK, Ellwein LB et al (2002) Refractive error in children in an urban population in New Delhi. Invest Ophthalmol Vis Sci 43:623–631 11867576

[14] Saw S-M, Tong L, Chua W-H et al (2005) Incidence and progression of myopia in Singaporean school children. Invest Ophthalmol Vis Sci 46:51–57. 10.1167/iovs.04-0565 15623754 10.1167/iovs.04-0565

[15] Ramamurthy D, Lin Chua SY, Saw S-M (2015) A review of environmental risk factors for myopia during early life, childhood and adolescence. Clin Exp Optom 98:497–506. 10.1111/cxo.12346 26497977 10.1111/cxo.12346

[16] Pan C-W, Qian D-J, Saw S-M (2017) Time outdoors, blood vitamin D status and myopia: a review. Photochem Photobiol Sci 16:426–432. 10.1039/C6PP00292G 27921098 10.1039/C6PP00292G

[17] Ashby RS, Schaeffel F (2010) The effect of bright light on lens compensation in chicks. Invest Ophthalmol Vis Sci 51:5247–5253. 10.1167/iovs.09-4689 20445123 10.1167/iovs.09-4689

[18] Ashby R, Ohlendorf A, Schaeffel F (2009) The effect of ambient illuminance on the development of deprivation myopia in chicks. Invest Ophthalmol Vis Sci 50:5348–5354. 10.1167/iovs.09-3419 19516016 10.1167/iovs.09-3419

[19] Jin J-X, Hua W-J, Jiang X et al (2015) Effect of outdoor activity on myopia onset and progression in school-aged children in northeast China: the Sujiatun Eye Care Study. BMC Ophthalmol 15:73. 10.1186/s12886-015-0052-9 26152123 10.1186/s12886-015-0052-9 PMC4495846

[20] Eppenberger LS, Sturm V (2020) The role of time exposed to outdoor light for myopia prevalence and progression: a literature review. Clin Ophthalmol 14:1875–1890. 10.2147/OPTH.S245192 32669834 10.2147/OPTH.S245192 PMC7337435

[21] He M, Xiang F, Zeng Y et al (2015) Effect of time spent outdoors at school on the development of myopia among children in China: a randomized clinical trial. JAMA 314:1142–1148. 10.1001/jama.2015.10803 26372583 10.1001/jama.2015.10803

[22] Bar Dayan Y, Levin A, Morad Y et al (2005) The changing prevalence of myopia in young adults: a 13-year series of population-based prevalence surveys. Invest Ophthalmol Vis Sci 46:2760–2765. 10.1167/iovs.04-0260 16043848 10.1167/iovs.04-0260

[23] McKnight CM, Sherwin JC, Yazar S et al (2014) Myopia in young adults is inversely related to an objective marker of ocular sun exposure: the Western Australian Raine cohort study. Am J Ophthalmol 158:1079–1085. 10.1016/j.ajo.2014.07.033 25072831 10.1016/j.ajo.2014.07.033 PMC4786165

[24] Jacobsen N, Jensen H, Goldschmidt E (2007) Prevalence of myopia in Danish conscripts. Acta Ophthalmol Scand 85:165–170. 10.1111/j.1600-0420.2006.00789.x 17305729 10.1111/j.1600-0420.2006.00789.x

[25] Midelfart A, Kinge B, Midelfart S, Lydersen S (2002) Prevalence of refractive errors in young and middle-aged adults in Norway. Acta Ophthalmol Scand 80:501–505. 10.1034/j.1600-0420.2002.800508.x 12390161 10.1034/j.1600-0420.2002.800508.x

[26] Sperduto RD, Seigel D, Roberts J, Rowland M (1983) Prevalence of myopia in the United States. Arch Ophthalmol 101:405–407. 10.1001/archopht.1983.01040010405011 6830491 10.1001/archopht.1983.01040010405011

[27] Bereit für die Rekrutierung? In: Schweizer Armee. https://www.vtg.admin.ch/de/mein-militaerdienst/stellungspflichtige/rekrutierung.html. Accessed 31 Aug 2023

[28] Militärdienst. https://www.ch.ch/de/sicherheit-und-recht/militardienst-und-zivildienst/militardienst#vor-dem-militardienst. Accessed 31 Aug 2023

[29] Yang L (2020) Thirty-five-year trend in the prevalence of refractive error in Austrian conscripts based on 1.5 million participants | Br J Ophthalmol. https://bjo.bmj.com/content/104/10/1338.long. Accessed 2 Oct 2022 10.1136/bjophthalmol-2019-315024 32024654

[30] Swiss Armed Forces Tauglichkeit der endgültig beurteilten Stellungspflichtigen 2016 nach Kantonen (Fitness for duty of conscripts finally assessed in 2016 by canton). https://www.newsd.admin.ch/newsd/message/attachments/47388.pdf. Accessed 13 Nov 2023

[31] Swiss Armed Forces Tauglichkeit der endgültig beurteilten Stellungspflichtigen 2014 nach Kantonen (Fitness for duty of conscripts finally assessed in 2014 by canton). https://www.newsd.admin.ch/newsd/message/attachments/38555.pdf. Accessed 13 Nov 2023

[32] Swiss Armed Forces Tauglichkeit der endgültig beurteilten Stellungspflichtigen 2013 nach Kantonen (Fitness for duty of conscripts finally assessed in 2013 by canton). https://www.newsd.admin.ch/newsd/message/attachments/34002.pdf. Accessed 13 Nov 2023

[33] Swiss Armed Forces Tauglichkeit der endgültig beurteilten Stellungspflichtigen 2012 nach Kantonen (Fitness for duty of conscripts finally assessed in 2012 by canton). http://www.news.admin.ch/NSBSubscriber/message/attachments/30661.pdf. Accessed 13 Nov 2023

[34] Swiss Armed Forces Tauglichkeit der endgültig beurteilten Stellungspflichtigen 2018 nach Kantonen (Fitness for duty of conscripts finally assessed in 2018 by canton). https://www.newsd.admin.ch/newsd/message/attachments/56291.pdf. Accessed 13 Nov 2023

[35] Swiss Armed Forces Tauglichkeit der endgültig beurteilten Stellungspflichtigen 2010 nach Kantonen (Fitness for duty of conscripts finally assessed in 2010 by canton). https://www.newsd.admin.ch/newsd/message/attachments/22160.pdf. Accessed 13 Nov 2023

[36] Swiss Armed Forces Tauglichkeit der endgültig beurteilten Stellungspflichtigen 2011 nach Kantonen (Fitness for duty of conscripts finally assessed in 2011 by canton). https://www.newsd.admin.ch/newsd/message/attachments/25854.pdf. Accessed 13 Nov 2023

[37] Swiss Armed Forces Tauglichkeit der endgültig beurteilten Stellungspflichtigen 2015 nach Kantonen (Fitness for duty of conscripts finally assessed in 2015 by canton). https://www.newsd.admin.ch/newsd/message/attachments/43136.pdf. Accessed 13 Nov 2023

[38] Swiss Armed Forces Tauglichkeit der endgültig beurteilten Stellungspflichtigen 2017 nach Kantonen (Fitness for duty of conscripts finally assessed in 2017 by canton). https://www.newsd.admin.ch/newsd/message/attachments/51588.pdf. Accessed 13 Nov 2023

[39] Swiss Armed Forces Tauglichkeit der endgültig beurteilten Stellungspflichtigen 2019 nach Kantonen (Fitness for duty of conscripts finally assessed in 2019 by canton). https://www.newsd.admin.ch/newsd/message/attachments/60357.pdf. Accessed 13 Nov 2023

[40] Hashemi H, Fotouhi A, Mohammad K (2004) The age- and gender-specific prevalences of refractive errors in Tehran: the Tehran Eye Study. Ophthalmic Epidemiol 11:213–225. 10.1080/09286580490514513 15370553 10.1080/09286580490514513

[41] Armarnik S, Lavid M, Blum S et al (2021) The relationship between education levels, lifestyle, and religion regarding the prevalence of myopia in Israel. BMC Ophthalmol 21. 10.1186/s12886-021-01891-w 10.1186/s12886-021-01891-w PMC7962316 33726690

[42] Schuster AK, Elflein HM, Pokora R, Urschitz MS (2017) Prevalence and risk factors of myopia in children and adolescents in Germany - results of the KiGGS survey. Klin Padiatr 229:234–240. 10.1055/s-0043-102938 28718190 10.1055/s-0043-102938

[43] Schuster AK, Krause L, Kuchenbäcker C et al (2020) Prevalence and time trends in myopia among children and adolescents. Dtsch Arztebl Int. 10.3238/arztebl.2020.0855 33612155 10.3238/arztebl.2020.0855 PMC8025934

[44] Sun J, Zhou J, Zhao P et al (2012) High prevalence of myopia and high myopia in 5060 Chinese university students in Shanghai. Invest Ophthalmol Vis Sci 53:7504–7509. 10.1167/iovs.11-8343 23060137 10.1167/iovs.11-8343

[45] Rudnicka AR, Kapetanakis VV, Wathern AK et al (2016) Global variations and time trends in the prevalence of childhood myopia, a systematic review and quantitative meta-analysis: implications for aetiology and early prevention. Br J Ophthalmol 100:882–890. 10.1136/bjophthalmol-2015-307724 26802174 10.1136/bjophthalmol-2015-307724 PMC4941141

[46] Population by migration status | Federal Statistical Office. https://www.bfs.admin.ch/bfs/en/home/statistics/population/migration-integration/by-migration-status.html. Accessed 17 Mar 2024

